# CS1, a controlled‐release formulation of valproic acid, for the treatment of patients with pulmonary arterial hypertension: Rationale and design of a Phase 2 clinical trial

**DOI:** 10.1002/pul2.12323

**Published:** 2024-01-03

**Authors:** Raymond L. Benza, Philip B. Adamson, Deepak L. Bhatt, Fredrik Frick, Gunnar Olsson, Niklas Bergh, Björn Dahlöf

**Affiliations:** ^1^ Ohio State Wexner Medical Center The Ohio State University Columbus Ohio USA; ^2^ Heart Failure Division Abbott Inc. Austin Texas USA; ^3^ Mount Sinai Heart Icahn School of Medicine at Mount Sinai New York New York USA; ^4^ Cereno Scientific Gothenburg Sweden; ^5^ Institute of Medicine University of Gothenburg Gothenburg Sweden; ^6^ Early Clinical Development, Biopharmaceuticals Research and Development—Cardiovascular Renal and Metabolism, AstraZeneca Mölndal Sweden

**Keywords:** CardioMEMS™, histone deacetylase inhibition, sodium valproate

## Abstract

Although rare, pulmonary arterial hypertension (PAH) is associated with substantial morbidity and a median survival of approximately 7 years, even with treatment. Current medical therapies have a primarily vasodilatory effect and do not modify the underlying pathology of the disease. CS1 is a novel oral, controlled‐release formulation of valproic acid, which exhibits a multi‐targeted mode of action (pulmonary pressure reduction, reversal of vascular remodeling, anti‐inflammatory, anti‐fibrotic, and anti‐thrombotic) and therefore potential for disease modification and right ventricular modeling in patients with PAH. A Phase 1 study conducted in healthy volunteers indicated favorable safety and tolerability, with no increased risk of bleeding and significant reduction of plasminogen activator inhibitor 1. In an ongoing randomized Phase 2 clinical trial, three doses of open‐label CS1 administered for 12 weeks is evaluating the use of multiple outcome measures. The primary endpoint is safety and tolerability, as measured by the occurrence of adverse events. Secondary outcome measures include the use of the CardioMEMS™ HF System, which provides a noninvasive method of monitoring pulmonary artery pressure, as well as cardiac magnetic resonance imaging and echocardiography. Other outcomes include changes in risk stratification (using the REVEAL 2.0 and REVEAL Lite 2 tools), patient reported outcomes, functional capacity, 6‐min walk distance, actigraphy, and biomarkers. The pharmacokinetic profile of CS1 will also be evaluated. Overall, the novel design and unique, extensive clinical phenotyping of participants in this trial will provide ample evidence to inform the design of any future Phase 3 studies with CS1.

## INTRODUCTION

Pulmonary arterial hypertension (PAH) is a rare disease, with a prevalence of approximately 10–15 cases per 100,000 individuals,^1^ a predominance of females and a mean age of 50–65 years at diagnosis.^2^, ^3^, ^4^ It has been suggested that the prevalence of PAH has increased over time.^5^

Although the median survival of patients with PAH has increased with treatment over the past 30 years,^6^, ^7^ it remains unacceptably low for a disease affecting primarily middle‐aged women, and survival rates have remained stable during the last decade.^4^, ^8^ Patients with PAH continue to live with substantial morbidity and impaired quality of life in spite of modern PAH‐specific treatments.^9^, ^10^, ^11^, ^12^

## RISK‐BASED MANAGEMENT OF PAH

As the progression of PAH and associated outcomes in a specific individual is unpredictable, risk stratification can support optimal management. Repeated risk assessments assessing the impact of medical therapy may facilitate tailoring and intensification of treatment.^13^ Most recently, the REVEAL 2.0 tool was developed to improve prediction of all‐cause mortality and morbidity due to complications (such as hospitalization) compared to the original REVEAL system.^14^, ^15^ This tool provides both categorical definitions of risk, that is, low, intermediate, and high scores and also continuous lines of risk scores.

A shorter version of REVEAL 2.0, REVEAL Lite 2, has been developed, which includes only six modifiable and noninvasive parameters and has been shown to approximate REVEAL 2.0 in terms of 1‐year mortality prediction.^16^ This tool can be used at every routine visit and gives an insight into disease trajectories that can prompt intervention. It is anticipated that in future clinical trials, changes in these risk scores could become a primary or surrogate outcome measure.^17^

## THE IMPORTANCE OF HEMODYNAMIC ASSESSMENT

PAH is hemodynamically defined based on elevated pulmonary artery (PA) mean pressure arising from decreased compliance and increased resistance to right ventricular (RV) outflow.^18^, ^19^ Chronic pulmonary resistance and decreased compliance eventually lead to RV dysfunction, which is associated with an exponentially increased risk of death.^20^, ^21^ Right heart catheterization (RHC) remains the gold standard for diagnosis of PAH. However, there are numerous disadvantages associated with repeated RHC, including invasiveness, risk of complications, and cost.^22^ Additionally, it provides only a snapshot of hemodynamic status at a single point in time, which may impair assessment of medication effectiveness.^23^

**Figure 1:**
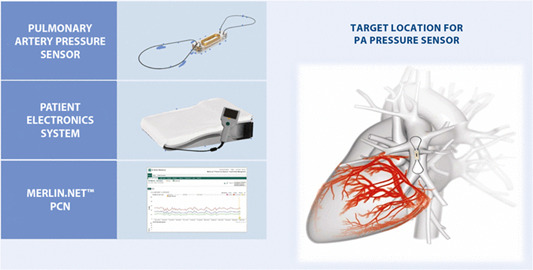
The CardioMEMS™ HF System. Permission to reproduce this image has been granted by Abbott, Inc.

The CardioMEMS™ HF System (Abbott Labs) (Figure 1) provides a microelectromechanical (MEMS) pressure sensor permanently implanted in the PA allowing remote monitoring of PA systolic, mean, and diastolic pressures along with heart rate on a daily basis to inform clinical decision‐making.^24^ The sensor does not have a lead or battery and is implanted in a distal branch of the PA during RHC.^25^ Patients activate the sensor using a home electronics unit which captures 18 s of PA pressure data as often as directed. Measured data are encrypted and uploaded to a secured website for healthcare provider review.

To date, it has been estimated that more than 30,000 patients are using the CardioMEMS system. In the pivotal CHAMPION trial, 550 patients with New York Heart Association (NYHA) Class III symptoms after a previous hospitalization underwent CardioMEMS implantation and were randomized to either the treatment group whose medical care was directed by PA pressure trends created by daily patient uploads or the control group whose uploaded pressures were not made available for medical decision‐making.^24^, ^26^ Of 575 patients, during 6 months of follow‐up 8 (1.4%) experienced device‐related or system‐related complications (defined as an adverse event [AE] that was definitely or possibly related to the wireless pressure sensor or external electronics, and was treated with invasive means other than intramuscular medication or RHC). No pressure sensor failures were reported.^26^ There were no additional complications of this nature observed during extended follow‐up (of at least 12 months).^27^ Hemodynamic‐guided heart failure management in the treatment group resulted in a 33% reduction in heart failure hospitalizations and a 16% reduction in all‐cause admissions. In another large study involving 1000 patients with NYHA Class II–IV symptoms (GUIDE‐HF) all subjects underwent CardioMEMS implantation followed by randomization to either PA pressure‐guided standard of care or standard of care only (guideline‐recommended titration of diuretics).^28^ At 12 months, 1% of patients experienced device‐related or system‐related complications (as defined in the CHAMPION study). Consistent reductions in rehospitalizations in patients treated with PA pressure guidance using CardioMEMS were observed. In a post‐approval study that enrolled 1200 patients with NYHA Class III symptoms, at 2 years of follow‐up, device‐ or system‐related complications occurred in 0.4% of patients and sensor failure in 0.1% of patients.^29^ Similar rates of device‐related or system‐related complications and sensor failure have been reported in other clinical trials and real‐world studies using CardioMEMS in heart failure patients.^30^, ^31^

Specific AEs observed in these studies have not been reported widely. In the MEMS‐HF study, one patient experienced a serious infection related to the device, and there were a small number of patients who experienced serious implant procedure‐related events, including infection, hemoptysis, bleeding, abnormal heart rate/rhythm, renal failure, and lead dislodgement/migration.^32^ In an analysis of data from the FDA Manufacturer and User Facility Device Experience Database, from a total of 2861 reports, hemoptysis was reported in 70 cases (2.4%), heart failure exacerbation in 43 cases (1.5%), and significant bleeding at the catheterization site in 24 cases (0.8%).^33^ CardioMEMS was associated with death or comfort care (undefined) in 167 (5.8%) of the reports.

To date, only one pilot study using CardioMEMS in 26 patients with NYHA Class III–IV PAH has been published.^34^ An experimental stroke volume algorithm was used in this study to further characterize the impact of PAH‐specific medications (vasodilators) on resistance and compliance measurements and was shown to correlate well with invasively measured volumes. Over a 1‐year period following implantation, there were no device‐related safety issues up to 12 months following implantation. Significant reductions in PA pressure and total pulmonary resistance were observed, accompanied by increases in cardiac output, RV stroke volume, and RV efficiency. With respect to clinical outcomes, there was an overall improvement in NYHA functional class, quality of life, and 6‐min walk distance (6MWD), due to the ability of CardioMEMS to guide dosing of medical therapies.^34^ It should be noted that the heart failure population differs from PAH patients in terms of the surrogate measures employed and the different medical therapies administered (diuretics vs. vasodilators).

These data support the hypothesis that PA pressure‐guided medical decision‐making may be safe in PAH patients requiring medical therapies. Additionally, more frequent and noninvasive PA pressure responses to medical intervention may provide an efficient and more accurate dosing evaluation for novel compounds addressing the basic mechanisms of PAH.

## EPIGENETIC MODIFICATION AS A TREATMENT MODALITY FOR PAH

The role of epigenetics in the pathogenesis of PAH has been under investigation for several years. Expression of specific histone deacetylases (HDACs) is known to be upregulated in the lung tissue, PA endothelial cells, remodeled pulmonary artery smooth muscle cells (PASMCs), and pulmonary artery adventitial fibroblasts (PAAFs) of patients with PAH.^35^, ^36^ Additionally, histone deacetylation is associated with decreased extracellular superoxide dismutase, an important antioxidant enzyme.^37^

**Figure 2:**
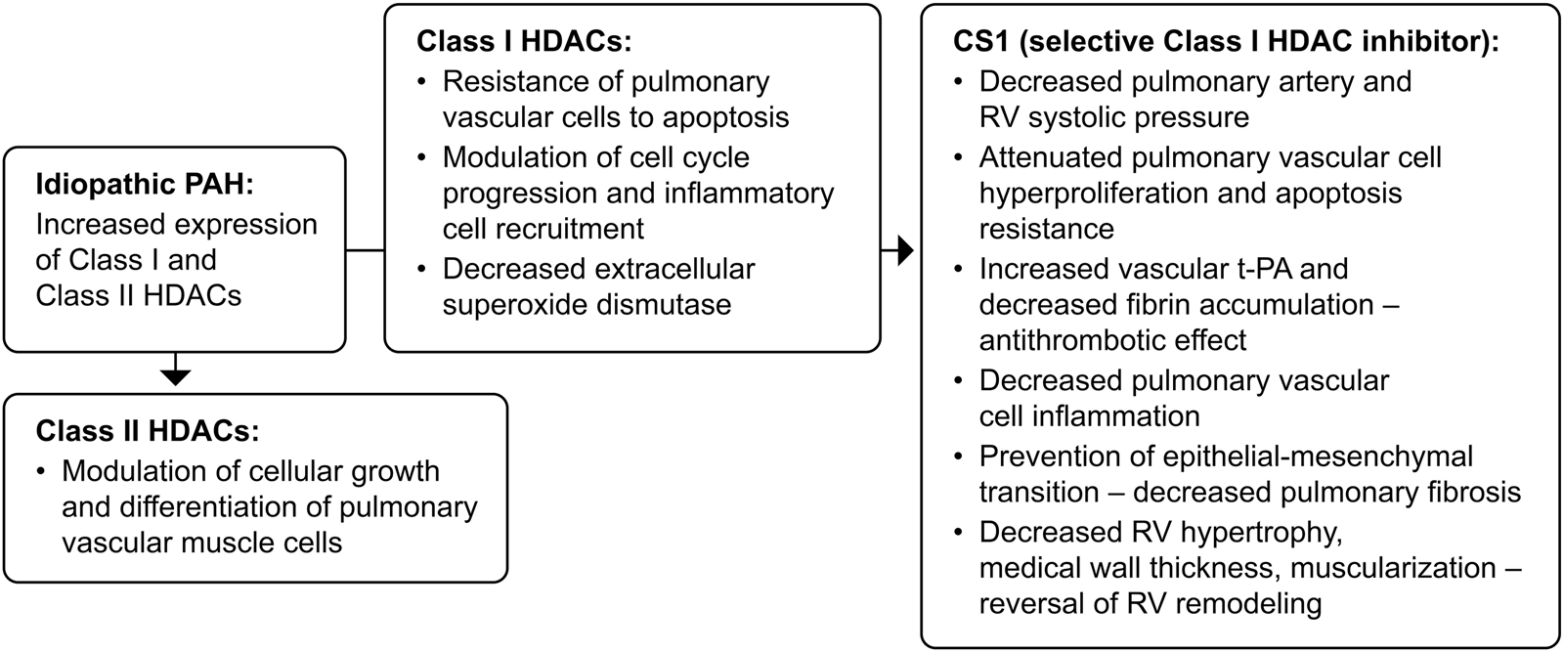
CS1 mode of action in PAH. HDAC, histone deacetylase; PAH, pulmonary arterial hypertension; RV, right ventricle; t‐PA, tissue plasminogen activator.

Valproic acid (VPA) is a selective Class I HDAC inhibitor that has shown activity in studies relevant to PAH (Figure 2). In models of severe pulmonary hypertension and in tissue from patients with PAH, VPA demonstrated significant reductions in RV systolic pressure, PA pressure, RV hypertrophy, medial wall thickness, muscularization, and vascular occlusion.^35^, ^36^, ^38^, ^39^ At the cellular level, VPA induces apoptosis in PAAFs and PASMCs from patients with PAH.^36^ VPA also has important anti‐inflammatory, anti‐fibrotic, and anti‐thrombotic properties, which may be advantageous in PAH.^39^, ^40^, ^41^ Therefore, VPA exhibits a unique multitargeted mechanism of action that allows for pressure reduction, reverse vascular remodeling, and positive RV adaptation.

CS1 is a novel oral controlled‐release formulation of sodium valproate in development for the treatment of PAH. Upon administration, the active substance is converted to VPA within the intestine. Dosing of CS1 (higher dose in the evening than in the morning) is designed to produce optimum VPA concentrations during the early morning hours when circulating plasminogen activator inhibitor 1 (PAI‐1) levels and the risk of thrombotic events are highest.^42^, ^43^ This differs from the typical evenly divided or once‐daily doses of sodium valproate used in the management of epilepsy.^44^, ^45^ In a Phase 1 clinical trial in healthy volunteers, CS1 demonstrated a good safety and tolerability profile alongside favorable pharmacokinetics (PK), and correlated with a significant reduction in plasminogen PAI‐1 levels as well as improved clot lysis times, with no increased bleeding risk.^46^ CS1 with its multitargeted mechanism of action has the potential to be a disease‐modifying therapy for PAH and should be evaluated in a well‐designed clinical trial.^17^

Here we present the design of a Phase 2 randomized clinical trial that will primarily evaluate the safety, tolerability, and exploratory efficacy of CS1 in patients with PAH. This trial utilizes a broad spectrum of contemporary and novel clinical assessments coupled with implantable PA pressure monitoring using the CardioMEMS HF System. Patient‐reported outcomes and other measures will facilitate high‐grade clinical phenotyping, which will then be utilized for subsequent high‐level Phase 3 planning.

## METHODS

### Study design

**Figure 3:**
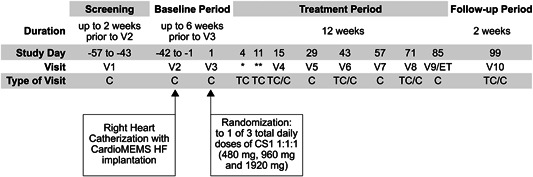
Study design schematic. C, clinic visit; ET, early termination; TC, telephone or video call; V, visit.

The Phase 2 prospective, randomized, parallel group, open‐label treatment, blinded endpoint multicenter study (design as per PROBE)^47^ (ClinicalTrials.gov identifier NCT05224531) is being conducted in several specialist PAH centers in the United States (listed at the end of this article). The study period is 22 weeks, including: a screening period of up to 2 weeks before the start of the baseline period, a baseline period of up to 6 weeks before randomization, a treatment period of 12 weeks, and a follow‐up period of 2 weeks (Figure 3).

Following screening, subjects undergo RHC with classical measurements and have a CardioMEMS PA Sensor implanted according to published methods,^24^, ^26^ followed by the baseline period for subjects to become familiar with the system, its measurements, how to send the data collected, and to establish a true ambulatory baseline PA pressure profile. During the study, mean PA pressure (mPAP) and other hemodynamic parameters from the CardioMEMS PA Sensor are measured and data captured once daily in the morning while the participant is in the supine position. Data are transferred electronically to a secured web‐based data repository.

The study is conducted in accordance with the ethical principles of the Declaration of Helsinki and International Conference on Harmonization Good Clinical Practice guidelines. The study protocol (version 3.0; December 9, 2022) was approved by the ethics committees at all participating study sites. CardioMEMS HF was approved for all forms of heart failure by the United States Food and Drug Administration (FDA) in 2014.^48^

### Study treatment

Participants are randomized 1:1:1 to one of three CS1 dose levels, with a target of 10 participants per dose level: 480 mg/day (1 × 160 mg capsule in the morning and 2 × 160 mg capsules in the evening); 960 mg/day (1 × 160 mg capsule in the morning and 2 × 160 mg capsules in the evening for 3 days; if tolerated this is followed by 2 × 160 mg capsules in the morning and 4 × 160 mg capsules in the evening for the remainder of the study); 1920 mg/day (1 × 160 mg capsule in the morning and 2 × 160 mg capsules in the evening for 3 days; if tolerated this is followed by 2 × 160 mg capsules in the morning and 4 × 160 mg capsules in the evening for 7 days; if tolerated this is increased on Day 11 to 4 × 160 mg capsules in the morning and 8 × 160 mg capsules in the evening for the remainder of the study). If the dose of CS1 following an increase is not tolerated, the dose is reduced. If the participant cannot tolerate the reduced dose they are withdrawn from the study but continue to be evaluated. No dose escalation is planned as the effect of CS1 on outcome measures other than safety and tolerability will not be evident until the study has concluded, and all participants are already receiving standard of care therapy. CS1 is formulated as controlled‐release capsules with mini‐tablets designed to release the active substance with a peak 10 h after administration containing in total 160 mg sodium valproate, which was manufactured by Galenica AB, for further details see the Phase 1 study publication.^46^ Participant number assignment and randomization will be conducted via use of an interactive response technology system at the screening and randomization visits.

No placebo group was deemed necessary for assessment of safety and tolerability as VPA has a well‐known safety and tolerability profile from previous studies. The FDA had no requirement for a placebo group for a conclusive study and advised that the study had an adequate design both at pre‐investigational new drug (IND) discussions and when the final IND was submitted. The placebo effect for subjective endpoints, for example, 6MWD are well‐known from many PAH studies in similar populations. Most of the key secondary endpoints from CardioMEMS, RHC, pulmonary vascular resistance (PVR), echocardiography, cardiac magnetic resonance imaging (MRI), and biomarkers, and so forth are unlikely to have placebo effects. The recent PULSAR study with sotatercept showed a minimal and nonsignificant placebo effect with PVR as measured using RHC.^49^

Dose selection was based on animal studies in established models for the treatment and prevention of PAH.^35^, ^39^ The effective doses of VPA in these studies was equivalent to a human dose of 1120–3430 mg/day, which was calculated using the published guidance from the FDA.^50^ Therefore, the current study evaluates a dose in the lower range (480 mg/day) which was well tolerated in the Phase 1 study in which CS1, at a dose of 552 mg/day, reduced PAI‐1 significantly,^46^ and was the dose closest to the lowest effective dose in animals (960 mg/day) and a dose in the higher dose range (1920 mg/day). The two higher doses evaluated are higher than those administered in the Phase 1 healthy volunteer study to allow further evaluation of the dose response in PA pressure reduction, vascular remodeling, anti‐inflammatory, anti‐fibrotic, and anti‐thrombotic effects. VPA has been used in the treatment of epilepsy for many years at higher doses than those evaluated in the Phase 1 study, so determination of the maximum tolerated dose/dose‐limiting toxicities were deemed unnecessary. CS1 is administered in unevenly divided daily doses with the larger portion given in the evening to minimize the risk of AEs and maximize the effect on endogenous PAI‐1 levels, which are higher in the early morning hours.^42^

### Outcome measures

The primary objective of the study is to evaluate the safety and tolerability of three doses of CS1, with secondary objectives of exploratory efficacy, measures of PAH progression and PK of CS1. For a full description of the primary and secondary outcomes see Tables 1 and 2.

**Table 1:** CS1 Phase 2 study endpoints and outcome measures.

*Primary endpoint*	
Safety outcomes	Adverse events Adverse events of special interest Serious adverse events Adverse device events related to the CardioMEMS HF System, including adverse device events, serious adverse device events, and unanticipated adverse device events Laboratory parameter abnormalities Change in vital signs Bleedings Change in ECG parameters
*Exploratory endpoints*	
Change from baseline to the end of treatment in right heart catheterization parameters—comparison between CS1 doses	PVR, PVRI, SVR, SVRI, PVRI:SVRI ratio, cardiac output and index, SV, SVI, RAP, right ventricular end diastolic pressure, PA systolic, diastolic and mean pressure, PCWP, RAP/PCWP, pulmonary compliance, pulmonary elastance, cardiac efficiency, cardiac power
REVEAL 2.0—comparison between CS1 doses	Score, strata, mean, and number of participants with reduction in score of ≥1 point at each visit, percentage of participants with change to low‐risk status (score <6), percentage of participants with change to ultra‐low‐risk status (score <5), percentage of participants with change to and maintenance of high‐risk status, percentage of participants with change to and maintenance of intermediate risk status
REVEAL Lite 2—comparison between CS1 doses	Score, strata, mean, and number of participants with reduction in score of ≥1 point at each visit, percentage of participants with change to REVEAL Lite 2 score <5, percentage of participants with change to REVEAL Lite 2 score <4, percentage of participants with change to and maintenance of high‐risk status, percentage of participants with change to and maintenance of intermediate risk status
Change from baseline to the end of treatment in patient‐reported outcomes—comparison between CS1 doses	Pulmonary Arterial Hypertension‐Symptoms and Impact Questionnaire Minnesota Living with Heart Failure® Questionnaire
Other clinical outcomes—comparison between CS1 doses	Need for additional inhaled or intravenous therapy throughout the study Change from baseline in NYHA/WHO functional class Number of all‐cause, pulmonary hypertension‐related, and cardiac (stroke, myocardial infarction, atrial fibrillation, heart failure of all types), venous thromboembolism hospitalizations, and hospitalizations for other reasons Number of deaths due to PAH, stroke, myocardial infarction, atrial fibrillation, heart failure (of all types), venous thromboembolism or other causes Registration for lung transplantation or atrial septostomy Change from baseline to end of treatment in 6MWD Change from baseline to end of treatment in eGFR
Change from baseline to the end of treatment in actigraphy measurements—comparison between CS1 doses	Percent increase in moderate activity, change in vector magnitude counts per minute, change in physical activity intensity, change in Daily Life Physical Activity spent in non‐sedentary activity, daily time spent in moderate‐to‐vigorous physical activity (min), number of steps
Change from baseline to the end of treatment in biomarkers—comparison between CS1 doses	BNP, NT‐pro‐BNP levels Suppression of tumorgenicity 2 levels Plasminogen activator inhibitor 1 levels Exploratory biomarkers
Pharmacokinetic endpoint—comparison between CS1 doses	Plasma VPA concentrations at study visits 4, 5, and 9 Steady‐state levels, *C*_max_, *T*_max_, AUC

Abbreviations: 6MWD, 6‐min walk distance; AUC, area under the concentration–time curve; BNP, brain natriuretic peptide; *C*_max_, maximum plasma concentration; ECG, electrocardiogram; eGFR, estimated glomerular filtration rate; NT‐pro‐BNP, N‐terminal‐pro‐brain natriuretic peptide; NYHA, New York Heart Association; PA, pulmonary artery; PAH, pulmonary arterial hypertension; PCWP, pulmonary capillary wedge pressure; PVR, pulmonary vascular resistance; PVRI, pulmonary vascular resistance index; RAP, right atrial pressure; SV, stroke volume; SVI, stroke volume index; SVR, systemic vascular resistance; SVRI, systemic vascular resistance index; *T*_max_, time to maximum plasma concentration; VPA, valproic acid; WHO, World Health Organization.

**Table 2:** CS1 Phase 2 study blindly read exploratory outcomes.

Change from baseline to the end of treatment in CardioMEMS PA Sensor measurements—comparison between CS1 doses	PA systolic, diastolic, and mean pressure, TPR, SV, SVI, pulmonary compliance, pulmonary elastance, cardiac efficiency, cardiac power, cardiac output and index, RVSW, RVSWI, RV efficiency (SV/mPAP)
Change from baseline to the end of treatment in cardiac MRI parameters—comparison between CS1 doses	PA sizes, right atrial area, RVEF, LVEF, SV, SVI, RV end diastolic and end systolic volume index, LV end diastolic and end systolic volume index, RV end diastolic volume:LV end diastolic volume ratio, pericardial effusion, RV end diastolic area, RV end systolic area, RV mass/index, PA relative area change, repetition time (TR; mild/moderate/severe), RV/PA coupling in relation to CardioMEMS
Change from baseline to the end of treatment in echocardiogram parameters—comparison between CS1 doses	RV end diastolic dimension, RV end systolic area, RV fractional area change, TAPSE, right atrial area, RV global, basal strain (TDIs’), systolic PAP, TAPSE/systolic PAP, RV global strain/systolic PAP, pericardial effusion, RV velocity time integral, PA acceleration time, TR (mild/moderate/severe)

Abbreviations: LV, left ventricle; LVEF, left ventricle ejection fraction; MRI, magnetic resonance imaging; PA, pulmonary artery; PAP, pulmonary artery pressure; RV, right ventricle; RVEF, right ventricle ejection fraction; RVSW, right ventricle stroke work; SV, stroke volume; SVI, stroke volume index; TAPSE, tricuspid annular plane systolic excursion; TPR, total pulmonary resistance; TR, repetition time.

The primary endpoint of the study includes AEs, AEs of special interest (liver toxicity, abnormal bleeding, anemia, thrombocytopenia, pancreatitis, tinnitus, dizziness, depression, and suicidal thoughts), serious AEs, adverse device effects related to the CardioMEMS HF System (adverse device effects, serious adverse device effects, and unanticipated adverse device effects, including those resulting from insufficient or inadequate instructions for use, deployment, implantation, installation, or operation, or any malfunction), laboratory parameter abnormalities, changes in vital signs, bleeding events and changes in ECG parameters (Table 1).

A number of exploratory efficacy endpoints are also planned, including changes from baseline in CardioMEMS PA Sensor hemodynamic measures, cardiac MRI parameters, and echocardiogram measures, in which the readers are blinded to participant randomization dose (Table 2). Other exploratory endpoints include RHC parameters, REVEAL 2.0 status, REVEAL Lite 2 status, NYHA/WHO functional class, 6MWD, estimated glomerular filtration rate (eGFR), actigraphy, and need for additional intravenous/inhaled therapy (Table 1). Pulmonary hypertension‐, cardiac‐ and venous thromboembolism‐related hospitalizations and deaths and need for lung transplantation or atrial septostomy are also exploratory endpoints. Biomarker assessments include brain natriuretic peptide (BNP), N‐terminal‐pro‐brain natriuretic peptide (NT‐pro‐BNP), serum tumorgenicity 2 (ST‐2), and PAI‐1.

Patient‐reported outcomes include change from baseline to the end of treatment in the Pulmonary Arterial Hypertension‐Symptoms and Impact Questionnaire scale and the Minnesota Living with Heart Failure® Questionnaire.

Plasma concentrations of VPA are measured at Visits 4, 5, and 9. At Visits 4 and 5, samples are obtained before the morning dose of CS1. At Visit 9, participants receive their final dose of CS1 and blood samples are obtained predose and 2, 4, 6, 8, 10, and 12 h after the morning dose. Maximum plasma concentration (*C*_max_), time to maximum plasma concentration (*T*_max_), and area under the concentration‐time curve (AUC) will be determined.

### Participants

Full details of inclusion and exclusion criteria are presented in Table 3. Male and female participants aged 18–50 years are eligible for the study if they have PAH as defined by National Institute of Care and Health Excellence,^51^ including idiopathic PAH, heritable PAH, drug/toxin‐induced PAH, or PAH associated with connective tissue disease. Subjects are required to have symptomatic PAH with a reduced exercise capacity related to PAH (NYHA/WHO functional class II/III; 6MWD ≥150 m and <550 m) and a REVEAL 2.0 score of 6–10. An RHC within the 36 months before study enrollment to confirm precapillary disease, with a resting mPAP ≥25 mmHg and resting mean PVR ≥5 Wood units with precapillary wedge pressure (PCWP) ≤ 15 mmHg. PAH therapy at stable doses of standard of care treatments for at least 90 days before screening is also required. Subjects with pulmonary hypertension category 2–5 and those likely to undergo lung transplantation within the next 6 months are excluded from the study. Participants are also ineligible to take part in the study if the CardioMEMS PA Sensor cannot be implanted due to active infection, recurrent thrombosis, intolerance of RHC, PA branch diameter <7 mm in the target implant vessel, or unable to take dual antiplatelet/anticoagulant therapy for 1 month after CardioMEMS PA Sensor implantation.

**Table 3:** Inclusion and exclusion criteria.

Inclusion criteria
1. Willing and able to sign a written informed consent before any study‐related procedures and able to understand and follow instructions; return to the study unit for specified study visits; and able to participate in the study for the entire period. 2. Male or females aged 18–80 years. 3. BMI of 18–40 kg/m^2^ at screening; if BMI is >35 kg/m^2^, subject chest circumference should be <65 inches (165 cm). 4. Subjects with PAH belonging to one of the following subgroups of NICE Clinical Classification of PAH category: a. Idiopathic PAH b. Heritable PAH c. Drug or toxin‐induced (anorexigen or methamphetamine use) d. PAH associated with connective tissue disease 5. Subjects with PAH who are symptomatic and have reduced exercise capacity due primarily to their PAH diagnosis, having been assessed by qualified individual, that is, physician, physician assistant, or nurse practitioner to be in NYHA/WHO functional class II or III and having a REVEAL 2.0 score of 6–10. 6. PAH therapy at stable doses of standard‐of‐care therapies for at least 90 days before screening. 7. Subject has most recent (within the last 36 months) hemodynamic assessment of PAH by RHC demonstrating a persistent resting mPAP ≥25 mmHg and resting mean pulmonary vascular resistance (PVR) ≥5 Wood Units with pulmonary capillary wedge pressure ≤15 mmHg. 8. Subject is willing to undergo CardioMEMS PA Sensor implantation and RHC before randomization or has had the CardioMEMS PA Sensor implanted previously. 9. 6MWD ≥150 and <550 m at screening 10. Female subjects of childbearing potential must be willing and able to practice effective contraception during the study and continuing contraception for 30 days after their last dose of study drug. Female subjects of non‐childbearing potential are defined as being surgically sterilized by bilateral tubal ligation, bilateral oophorectomy, or hysterectomy. A female subject 45–60 years of age, who is post‐menopausal for at least 1 year, and has a follicle‐stimulating hormone level confirmation indicating post‐menopausal status will be considered of non‐childbearing potential. Female subjects >60 years of age are considered post‐menopausal and of non‐childbearing potential.
Exclusion criteria 1. Pulmonary hypertension category 2–5. 2. Adult congenital heart disease (ACHD). 3. Concomitant medical or psychiatric disorder, condition, history, or any other condition that in the opinion of the Investigator would either put the subject at risk or impair the subject's ability to participate in or complete the requirements of the study or confound the objectives of the study. 4. A concomitant medical disorder that is expected to limit the subject's life‐expectancy to ≤1 year. 5. REVEAL 2.0 score of ≤5 or ≥11. 6. Heart failure with preserved ejection fraction defined as >50% ejection fraction (with signs and symptoms of heart failure) or left atrial volume >34 mL/m^2^. 7. Subject is not able to have CardioMEMS HF System implanted due to: a. An active, ongoing infection defined as being febrile, an elevated white blood cell count, on intravenous antibiotics, and/or positive cultures (blood, sputum, or urine). b. History of current or recurrent (≥2 episodes within 5 years before consent) pulmonary emboli and/or deep vein thromboses. c. Cannot tolerate RHC. d. PA branch inner diameter <7 mm in a descending branch within the left or right lower lung lobe (target implant vessel). e. Unable to take dual antiplatelet or anticoagulation therapy for 1 month after CardioMEMS implantation. 8. Likely to undergo lung transplantation within the next 6 months. 9. Untreated, moderate to severe obstructive sleep apnea. 10. Evidence of significant chronic thromboembolic disorder as determined by the Investigator or recent pulmonary embolism within 6 months before screening. 11. Uncontrolled hypertension (˃160/100 mmHg, confirmed by duplicate seated readings) at two or more historical visits within 3 months before screening. 12. Sustained systolic blood pressure <95 mmHg and/or diastolic blood pressure <50 mmHg (confirmed by duplicate seated readings) on at least three consecutive occasions (self‐monitored or office) before or at screening, or overt symptomatic hypotension. 13. Sustained resting heart rate >120 beats per minute (confirmed by duplicate assessments of office vital signs) or consecutive ECG assessments on at least three consecutive occasions before or at screening. 14. A history of a bleeding disorder. 15. Thrombocytopenia (platelets <150,000/mm^3^). 16. Known porphyria, mitochondrial disease, or urea cycle disease. 17. A history of chronic pancreatic disease. 18. Pregnant or lactating. 19. A positive result from serology testing at screening for HIV, HBsAg, HCV; but if the subject has a historical diagnosis (before screening) of being positive for HIV, HBsAg, or HCV must be clinically stable and if on therapy, be on stable therapy for ≥3 months before screening. A subject should not have active COVID‐19; however, those with previous COVID‐19 are permitted. 20. Participation in another investigational drug study within 30 days before screening or participating in a non‐medication study which, in the opinion of the Investigator, would interfere with the study compliance or outcome assessments. 21. Subject is on regular treatment with VPA, other antiseizure medications, or other prohibited medications that cannot be discontinued at the screening visit. 22. Regular anticoagulation or DAPT that cannot be discontinued at the screening visit; however, during the baseline period following CardioMEMS implantation, DAPT is allowed according to clinical practice for up to 4 weeks. 23. More than mild mitral or aortic valve disease, LVEF <50%, or left ventricular regional wall motion abnormality suggestive of active coronary artery disease on two‐dimensional‐echocardiogram at screening. 24. Subject has a forced expiratory volume in 1 s (FEV1)/forced vital capacity <70% (absolute), FEV1 ≤50% or total lung capacity (TLC) <70% predicted on pulmonary function testing (PFT); for potential subjects with TLC 60%–69% predicted, non‐contrasted computerized tomography (CT) scan must be performed to exclude subjects with more than mild interstitial lung disease. PFTs should have been obtained within 3 years before screening. 25. Clinically significant renal dysfunction (eGFR of <30 mL/min/1.73 m^2^) as calculated by Modification of Diet in Renal Disease (MDRD) at screening. 26. Significant liver dysfunction as measured by any one of the following at screening (including subjects with acute or chronic hepatitis as well as subjects with own or family history of serious hepatitis, especially drug related): a. ALT >2.0 × ULN. b. AST >2.0 × ULN. c. Serum bilirubin ≥1.6 mg/dL or >2.0 × ULN. 27. A known history of substance abuse including alcohol abuse within the 1 year before screening that in the opinion of the Investigator would impair the subject's ability to participate in or complete the requirements of the study. 28. Any major surgical procedure or trauma within 30 days before screening or planned surgical procedure during the study period. 29. Any inpatient hospitalization (defined as >23 h) within 30 days before screening. 30. Enrollment within 90 days before screening or plans to enroll during the study into a cardiopulmonary rehabilitation program. 31. Known hypersensitivity to study drug or any of the excipients of the drug formulation.

Abbreviations: 6MWD, 6‐minute walk distance; ALT, alanine aminotransferase; AST, aspartate aminotransferase; BMI, body mass index; DAPT, dual antiplatelet therapy; eGFR, estimated glomerular filtration rate; HBsAg, hepatitis B surface antigen; HCV, hepatitis C; HIV, human immunodeficiency virus; LVEF, left ventricular ejection fraction; mPAP, mean pulmonary artery pressure; NICE, National Institute of Health and Care Excellence; NYHA, New York Heart Association; PA, pulmonary artery; PAH, pulmonary arterial hypertension; PVR, pulmonary vascular resistance; RHC, right heart catheterization; ULN, upper limit of normal; VPA, valproic acid; WHO, World Health Organization.

### Sample size calculation

The main purpose of the study is to evaluate safety/tolerability of three different doses of CS1. In addition, exploratory evaluations of many relevant measurements for patients with PAH (see previous text sections) will be performed. For sample size calculation for the study, the estimate was based on the potential effect of CS1 on mPAP, with analysis according to multiple readings that increase the precision of PA pressure determination. A sample size of 10 subjects per dose level group has an 80% power to detect a difference in means of 15%, assuming that the common standard deviation is 5, with a 5% significance level.

PA pressure (mPAP) AUC will be the first secondary endpoint that will be tested statistically since the power of the study is based on that variable.

### Statistical analysis

The study protocol and Statistical Analysis Plan will be published as appendices to the primary publication following the end of the study.

The Safety Analysis Set includes all randomly assigned participants who receive at least one dose of study drug and participants will be analyzed as treated. The PK Analysis Set will include all participants in the Safety Analysis Set who have at least one evaluable post‐baseline PK sample, and this will be used for all PK analyses.

All data listings, summaries, figures, and statistical analyses will be generated using SAS version 9.4. Summaries will be presented by dosing cohort and overall. The exploratory efficacy endpoints will summarized descriptively and analyzed using both intention to treat as well as per protocol approaches. Of the exploratory efficacy endpoints, mPAP which the study is powered for, will be tested first to detect mean differences among dosing groups. Descriptive statistics for categorical variables will include the number and percentage of participants with each characteristic. Percentages will be based on the number of participants with non‐missing values. Descriptive statistics for ordinal and continuous variables will include the number of participants, mean, median, standard deviation, minimum value, maximum value.

## DISCUSSION

PAH remains a serious condition with great unmet need due to its incurable nature and significant associated morbidity and mortality. Currently approved therapies do not fully modify disease pathology and new treatments are therefore needed.

VPA has a multitude of effects that are amenable to treatment of PAH, including reverse remodeling of the underlying vascular changes, reduction of PA pressure, fibrosis, inflammation and thrombosis.^35^, ^36^, ^38^, ^39^ Sotatercept, which acts to reverse remodeling and reduce inflammation,^52^, ^53^ is efficacious in patients with PAH.^54^ Additionally, a meta‐analysis has shown that anticoagulation is associated with improved survival in patients with idiopathic PAH.^55^ CS1 therefore represents a novel approach to the management of PAH with a disease‐modifying potential.

Design of clinical trials is important to fully understand the mechanisms and benefits of new treatments. 6MWD has long been a fundamental outcome measure in studies investigating the effects of treatment in patients with PAH. However, a statistically significant change in 6MWD is not always clinically meaningful.^56^ More recently, composite endpoints encompassing both morbidity and mortality have been employed in clinical trials.^57^, ^58^ Time to clinical worsening (TTCW) is now a more widely used measure; however, there is no universal definition of TTCW, which makes comparisons between different clinical trials challenging.^17^ The use of composite endpoints also poses a further question regarding weighting of the individual components according to clinical importance and the frequency at which they occur.^59^ Further research is required to fully establish TTCW as a validated surrogate outcome for mortality in trials evaluating treatments.

Numerous potential pharmacological treatments for PAH have shown promise in preclinical models but have failed to show clinical benefit in humans or were associated with poor tolerability.^59^ There is an inherent issue with the use of preclinical models in that they do not closely approximate the disease in humans and typically do not involve background therapy, which is common in PAH patients. Furthermore, there is an unknown factor for potential off‐target effects of novel therapies. Refinement of preclinical testing is therefore needed to reduce false‐positive outcomes.

It has been suggested that the focus of early‐phase clinical trials in patients with PAH should be safety, determination of predictive biomarkers and identification of responders to treatment, while later‐phase trials should assess dose–response relationships and characterization of these responsive individuals.^59^ In the CS1 Phase 2 study, a wide array of functional, hemodynamic, and structural biomarkers as well as patient‐reported outcomes are being measured to provide an insight into not only treatment effects but the disease process itself. The use of highly discriminatory and calibrated risk stratification tools such as REVEAL 2.0 and REVEAL Lite 2 may also aid recruitment of a more homogeneous study population that may truly benefit from target drug application, i.e., enrichment. It has been shown that PAH patients who maintain a low‐risk status after initial treatment fare better than those who fall into the intermediate‐ and high‐risk categories^14^, ^60^, ^61^ and this also represents a valuable assessment of clinical worsening or improvement. In the FREEDOM‐EV study, a 1‐point decrease from baseline at Week 12 in REVEAL 2.0 score predicted a 62% reduction in the relative risk of clinical worsening, while a 1‐point decrease in REVEAL Lite 2 score predicted a 59% reduction in the relative risk of clinical worsening.^62^ The use of patient‐reported outcome measures (such as the Pulmonary Arterial Hypertension‐Symptoms and Impact Questionnaire and Minnesota Living with Heart Failure® Questionnaire included in the current study) also provide an insight into treatment effects distinct from those that are purely clinical or mechanistic.

Understanding hemodynamics at baseline and changes over time is also crucial to guide clinical management of patients with PAH. Notably, the recent Phase 2 clinical trial of sotatercept in PAH employed PVR as its primary endpoint, which was significantly different when comparing active treatment to placebo.^49^ It has been shown that the CardioMEMS HF System permits timely and consistent PA pressure monitoring that is associated with a more frequent medication changes and reduced hospitalizations in patients with heart failure regardless of left ventricular ejection fraction.^26^, ^31^, ^32^, ^34^, ^63^, ^64^, ^65^ Ambulatory PA pressure monitoring using CardioMEMS provides very frequent hemodynamic assessment in the patients’ normal environment, which more accurately assesses medication impact over time. Intermittent RHCs provide limited “real‐time” information about medication changes given the time between tests. This approach allows medication intervention studies to provide clearer data with fewer participants enrolled. Its use in the current study will provide greater insight into the real‐time evolution and stability/consistency of effects of CS1 at the level of pulmonary hemodynamics, PA and RV interactions, as well as provide important safety data using CardioMEMS in patients with PAH.

It is well‐established that the use of cardiac MRI is a valuable predictive tool for clinical worsening and mortality in PAH, in terms of tracking deterioration in RV function.^66^, ^67^, ^68^ In this study, the extensive imaging data captured will allow a precise insight into the adaptive change in RV response to therapy, which has been shown previously in a study of macitentan.^69^

In conclusion, CS1 represents a potential novel disease‐modifying treatment for PAH. The Phase 2 study described here will provide vital information regarding the safety of CS1 at three dose levels but will also include a broad range of endpoints encompassing clinical, hemodynamic, and PK parameters as well as patient‐reported outcomes. Topline results from this study are expected in the first quarter of 2024 and a quality analysis will be reported in the fall of 2023.

## AUTHOR CONTRIBUTIONS

Raymond L. Benza, Niklas Bergh, and Björn Dahlöf designed the study under direction of the Steering Committee and all authors contributed to the development of the manuscript. All authors, with the exception of Niklas Bergh constitute the Steering Committee for the study.

## CONFLICTS OF INTEREST STATEMENT

Raymond L. Benza has participated in steering committees for Cereno, Liquidia, United therapeutics, Gossamer, Insmed, and Abbott and as a consultant for Cereno, Abbott, and Merck. Philip B. Adamson is the Chief Medical Officer and Divisional Vice President of Abbott's Heart Failure Division receiving salary support and stockholder. Björn Dahlöf is CMO, CSO, and head of Clinical development at Cereno Scientific and has shares and warrants (equity) in Cereno Scientific. Fredrik Frick is Head of Clinical Operations at Cereno Scientific and has warrants in Cereno Scientific. Niklas Bergh is a senior consultant at Cereno Scientific and has shares and warrants (equity) in Cereno Scientific. Deepak L. Bhatt discloses the following relationships—Advisory Board: Angiowave, Bayer, Boehringer Ingelheim, Cardax, CellProthera, Cereno Scientific, Elsevier Practice Update Cardiology, High Enroll, Janssen, Level Ex, McKinsey, Medscape Cardiology, Merck, MyoKardia, NirvaMed, Novo Nordisk, PhaseBio, PLx Pharma, Regado Biosciences, Stasys; Board of Directors: Angiowave (stock options), Boston VA Research Institute, Bristol Myers Squibb (stock), DRS.LINQ (stock options), High Enroll (stock), Society of Cardiovascular Patient Care, TobeSoft; Chair: Inaugural Chair, American Heart Association Quality Oversight Committee; Consultant: Broadview Ventures, Hims; Data Monitoring Committees: Acesion Pharma, Assistance Publique‐Hôpitaux de Paris, Baim Institute for Clinical Research (formerly Harvard Clinical Research Institute, for the PORTICO trial, funded by St. Jude Medical, now Abbott), Boston Scientific (Chair, PEITHO trial), Cleveland Clinic (including for the ExCEED trial, funded by Edwards), Contego Medical (Chair, PERFORMANCE 2), Duke Clinical Research Institute, Mayo Clinic, Mount Sinai School of Medicine (for the ENVISAGE trial, funded by Daiichi Sankyo; for the ABILITY‐DM trial, funded by Concept Medical), Novartis, Population Health Research Institute; Rutgers University (for the NIH‐funded MINT Trial); Honoraria: American College of Cardiology (Senior Associate Editor, Clinical Trials and News, ACC.org; Chair, ACC Accreditation Oversight Committee), Arnold and Porter law firm (work related to Sanofi/Bristol‐Myers Squibb clopidogrel litigation), Baim Institute for Clinical Research (formerly Harvard Clinical Research Institute; RE‐DUAL PCI clinical trial steering committee funded by Boehringer Ingelheim; AEGIS‐II executive committee funded by CSL Behring), Belvoir Publications (Editor in Chief, Harvard Heart Letter), Canadian Medical and Surgical Knowledge Translation Research Group (clinical trial steering committees), CSL Behring (AHA lecture), Cowen and Company, Duke Clinical Research Institute (clinical trial steering committees, including for the PRONOUNCE trial, funded by Ferring Pharmaceuticals), HMP Global (Editor in Chief, Journal of Invasive Cardiology), Journal of the American College of Cardiology (Guest Editor; Associate Editor), K2P (Co‐Chair, interdisciplinary curriculum), Level Ex, Medtelligence/ReachMD (CME Steering Committees), MJH Life Sciences, Oakstone CME (Course Director, Comprehensive Review of Interventional Cardiology), Piper Sandler, Population Health Research Institute (for the COMPASS operations committee, publications committee, steering committee, and USA national co‐leader, funded by Bayer), Slack Publications (Chief Medical Editor, Cardiology Today's Intervention), Society of Cardiovascular Patient Care (Secretary/Treasurer), WebMD (CME Steering Committees), Wiley (Steering Committee); Other: Clinical Cardiology (Deputy Editor), NCDR‐ACTION Registry Steering Committee (Chair), VA CART Research and Publications Committee (Chair); Patent: Sotagliflozin (named on a patent for sotagliflozin assigned to Brigham and Women's Hospital who assigned to Lexicon; neither I nor Brigham and Women's Hospital receive any income from this patent); Research Funding: Abbott, Acesion Pharma, Afimmune, Aker Biomarine, Alnylam, Amarin, Amgen, AstraZeneca, Bayer, Beren, Boehringer Ingelheim, Boston Scientific, Bristol‐Myers Squibb, Cardax, CellProthera, Cereno Scientific, Chiesi, CinCor, Cleerly, CSL Behring, Eisai, Ethicon, Faraday Pharmaceuticals, Ferring Pharmaceuticals, Forest Laboratories, Fractyl, Garmin, HLS Therapeutics, Idorsia, Ironwood, Ischemix, Janssen, Javelin, Lexicon, Lilly, Medtronic, Merck, Moderna, MyoKardia, NirvaMed, Novartis, Novo Nordisk, Otsuka, Owkin, Pfizer, PhaseBio, PLx Pharma, Recardio, Regeneron, Reid Hoffman Foundation, Roche, Sanofi, Stasys, Synaptic, The Medicines Company, Youngene, 89Bio; Royalties: Elsevier (Editor, Braunwald's Heart Disease); Site Co‐Investigator: Abbott, Biotronik, Boston Scientific, CSI, Endotronix, St. Jude Medical (now Abbott), Philips, SpectraWAVE, Svelte, Vascular Solutions; Trustee: American College of Cardiology; Unfunded Research: FlowCo, Takeda. Dr. Olsson is a senior consultant at Cereno Scientific and has warrants in Cereno Scientific.

## ETHICS STATEMENT

The study is conducted in accordance with the ethical principles of the Declaration of Helsinki and International Conference on Harmonization Good Clinical Practice guidelines. The study protocol (version 3.0; December 9, 2022) was approved by the ethics committees at all participating study sites.

## STUDY INVESTIGATORS

Dr. Hubert J. Ford, Department of Internal Medicine, Division of Pulmonary and Critical Care Medicine, University of North Carolina, Chapel Hill, North Carolina, USA. Dr. Jason Guichard, Prisma Health, Greenville, South Carolina, USA. Dr. Evelyn M. Horn, Department of Internal Medicine, Division of Cardiology, Weill‐Cornell Medical School, New York, New York, USA. Dr. Selim Krim, Ochsner Medical Center, Baton Rouge, Louisiana, USA. Dr. James Murphy, Heart Center, Huntsville Hospital, Huntsville, Alabama, USA. Dr. Sandhya Murthy, Division of Cardiology, Montefiore Medical Center, Albert Einstein College of Medicine, New York, New York, USA. Dr. Farhan Raza, Division of Cardiovascular Medicine, Department of Medicine, University of Wisconsin School‐Madison, Madison, Wisconsin, USA. Dr. Marc A. Simon, Division of Cardiology, Department of Internal Medicine, University of California San Francisco, San Francisco, California, USA. Dr. Veronica Franco, Department of Internal Medicine, Division of Cardiovascular Medicine, Davis Heart and Lung Research Institute, Ohio State University, Columbus, Ohio, USA.
